# Spinal Cord Blood Vessels in Aged Mice Show Greater Levels of Hypoxia-Induced Vascular Disruption and Microglial Activation

**DOI:** 10.3390/ijms241411235

**Published:** 2023-07-08

**Authors:** Sebok K. Halder, Richard Milner

**Affiliations:** San Diego Biomedical Research Institute, 3525 John Hopkins Court, Suite 200, San Diego, CA 92121, USA; shalder@sdbri.org

**Keywords:** spinal cord, aging, blood vessels, microglia, chronic mild hypoxia, blood–spinal cord barrier integrity, fibrinogen, angiogenesis, endothelial proliferation

## Abstract

In response to chronic mild hypoxia (CMH, 8% O_2_), spinal cord blood vessels launch a robust angiogenic response that is associated with transient disruption of the blood–spinal cord barrier (BSCB) which, in turn, triggers a microglial vasculo-protective response. Because hypoxia occurs in many age-related conditions, the goal of this study was to define how aging influences these responses by comparing events in young (8–10 weeks) and aged (20 months) mice. This revealed that aged mice had much greater (3–4-fold) levels of hypoxic-induced BSCB disruption than young mice and that, while the early stage of the angiogenic response in aged mice was no different to young mice, the maturation of newly formed vessels was significantly delayed. Interestingly, microglia in the spinal cords of aged mice were much more activated than young mice, even under normoxic conditions, and this was further enhanced by CMH, though, surprisingly, this resulted in reduced microglial clustering around leaky blood vessels and diminished vasculo-protection. Vascular disruption was associated with loss of myelin in spinal cord white matter (WM) in both young and aged mice. Furthermore, it was notable that the spinal cord of aged mice contained a lower density of Olig2+ oligodendroglial cells even under normoxic conditions and that CMH significantly reduced the density of Olig2+ cells in spinal cord WM of the aged, but not the young, mice. These results demonstrate that spinal cord blood vessels of aged mice are much more vulnerable to the damaging effects of hypoxia than young mice, in part due to the reduced vasculo-protection conferred by chronically activated microglial cells. These observations may have implications for the pathogenesis and/or treatment of spinal cord diseases such as amyotrophic lateral sclerosis (ALS) and suggest that an improvement in microglial function could offer therapeutic potential for treating these age-related conditions.

## 1. Introduction

Blood vessels in the central nervous system (CNS) are unique, not just in their structural and functional properties [1,2], but also in their incredible plasticity to mount a strong and vigorous remodeling response when confronted with conditions of low oxygen (hypoxia) [3,4]. It is well established that CNS blood vessels in the brain and spinal cord have specialized properties of low permeability and high electrical resistance, which helps to both protect the sensitive CNS cells from potentially deleterious components in the blood [1,2] while also selectively permitting the passage of specific metabolites (glucose, amino acids, fatty acids) required by CNS neural cells. The structural basis of this blood-brain barrier (BBB) resides at several levels, including the adherens and tight junction protein complexes, that form between adjacent endothelial cells, the extracellular matrix (ECM) proteins within the vascular basement membrane, as well as the influence of adjacent CNS cells such as astrocytes, pericytes, and microglia [2,5,6,7,8]. In the spinal cord, the BBB is more correctly referred to as the blood–spinal cord barrier (BSCB), and its breakdown occurs in several neurological conditions, including meningitis, multiple sclerosis (MS), spinal cord injury (SCI), and amyotrophic lateral sclerosis (ALS, also known as motor neuron disease or Lou Gehrig’s disease) [9,10,11,12].

In previous studies we showed that chronic mild hypoxia (CMH, 8% O_2_) triggers two important events in spinal cord blood vessels. On the plus side, CMH triggers a strong angiogenic response characterized by widespread endothelial proliferation that culminates in significant and robust increases in the vascular area of both white matter (WM) and grey matter (GM) [13]. Importantly, these changes are associated with marked upregulation of the endothelial tight junction proteins claudin-5, occludin, and ZO-1, suggesting that, after completion of the vascular remodeling program, the spinal cord blood vessels have greater vascular integrity. The downside is that, in the short term, CMH also induces transient vascular disruption, leading to the leakage of blood constituents into the tissue parenchyma [7]. Importantly, this vascular disruption is counterbalanced by a strong microglia vasculo-protective response in which quiescent microglia become activated to migrate and aggregate around leaky vessels and thereby promote vessel repair [7].

As hypoxia occurs in a wide variety of medical conditions, both in the young (asthma and sleep apnea) and much more in the aged (due to increased incidence of chronic obstructive pulmonary disease (COPD), cardiac, and cerebrovascular disease) [14,15,16,17,18], it is important to examine how these hypoxia-induced changes in the properties of spinal cord blood vessels are influenced by age. It is particularly interesting to study these events in spinal cord because this part of the CNS is cleanly demarcated into separate WM and GM compartments, each with their own unique vascular properties of vessel type, structure, and density. Notably, the vessel density of GM is much (approximately 4-fold) greater than WM and contains numerous wide-caliber blood vessels of both arterial and venous type, while WM vessels are almost entirely small caliber capillaries [13,19,20]. Interestingly, our prior studies showed that the WM mounts a much stronger vascular remodeling response to hypoxia because, despite having only 25% the vessel density of GM, the levels of endothelial proliferation and vascular leak were at least as high, if not greater than those in GM [13]. Based on these considerations, the overall goal of this study was to build on our previous observations by addressing the following questions: (i) how does age influence hypoxia-induced vascular remodeling and BSCB disruption in the spinal cord, (ii) are there critical differences in these responses between the WM and GM compartments, and (iii) how do hypoxia and age affect the integrity of WM myelin and cells of the oligodendroglial lineage?

## 2. Results

### 2.1. Hypoxia-Induced Vascular Disruption in the Spinal Cord Is Markedly Increased in Aged Mice

**Figure 1:**
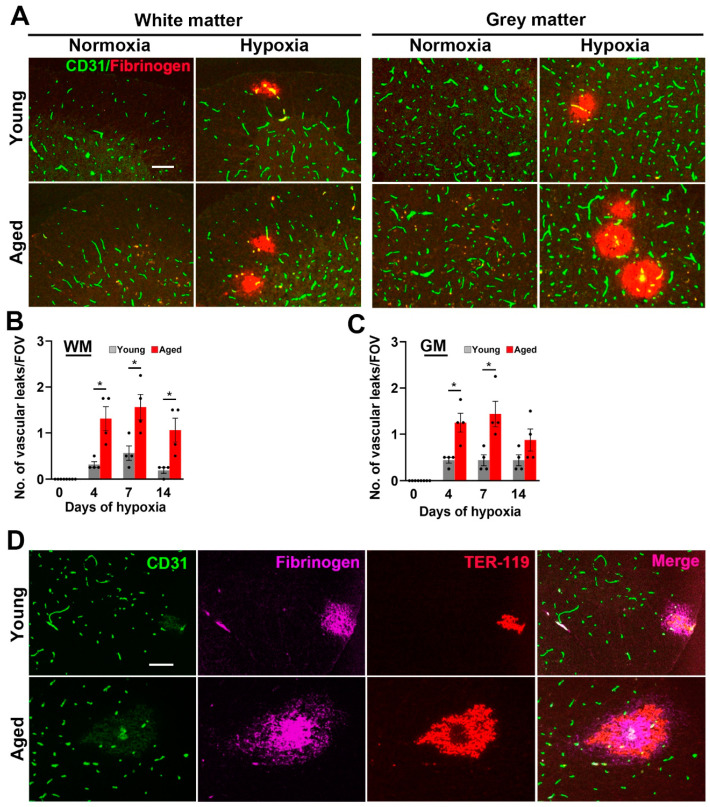
Chronic mild hypoxia (CMH)-induced vascular leak is greater in the aged spinal cord. (**A**) Frozen spinal cord sections taken from young (8–10 weeks) or aged (20 months) mice exposed to normoxia or 7 days hypoxia (8% O_2_) were stained for the endothelial marker CD31 (AlexaFluor-488) and fibrinogen (Cy-3). Scale bar = 100 μm. (**B**,**C**) Quantification of the number of vascular leaks (fibrinogen-positive)/FOV in spinal cord white matter (WM; panel **B**) and grey matter (GM; panel **C**) after 0, 4, 7, and 14 days hypoxia. All results are expressed as the mean ± SEM (*n* = 4 mice/group). * *p* < 0.05 vs. normoxic conditions. Note that vascular leak was significantly greater in aged spinal cord (both WM and GM) and that the highest number of vascular leaks peaked after 7 days hypoxia but declined by day 14. (**D**) Frozen brain sections taken from mice exposed to 7 days hypoxia (8% O_2_) were triple-labelled for CD31 (AlexaFluor-488), fibrinogen (Cy-5), and the red blood cell marker TER-119 (Cy-3). Scale bar = 100 μm. Note that many of the fibrinogen+ leaks also stained positive for TER-119, demonstrating the presence of hemorrhage in these regions.

We previously demonstrated that chronic mild hypoxia (CMH, 8% O_2_ for periods up to 14 days) triggers vascular disruption in the spinal cord [7]. To evaluate how this process is influenced by age, we compared levels of vascular disruption in young (8–10 weeks) and aged (20 months) female C57BL6/J mice by dual-immunofluorescence (dual-IF) of frozen spinal cord sections, using CD31 to label blood vessels and fibrinogen to identify extravascular leak. As shown in Figure 1A, under normoxic conditions, no vascular disruption was observed in spinal cord blood vessels, either in white matter (WM) or grey matter (GM) in young or aged mice. By contrast, in young mice, CMH triggered vascular disruption in a small number of blood vessels both in WM and GM and, strikingly, this number was significantly increased in aged mice (*p* < 0.05 after 4 or 7 days CMH, both in WM and GM; Figure 1A–C). Consistent with our previous observations [7], the number of vascular leaks was highest at the 7 day timepoint, but had declined by the 14 day timepoint. In addition to using extravascular fibrinogen leak to detect vascular breakdown, we also stained for the red blood cell marker, TER-119 (Figure 1D). This revealed that many of the fibrinogen+ leaks also stained positive for TER-119, thus demonstrating the presence of hemorrhage in these regions.

### 2.2. Age Has No Impact on Hypoxia-Induced Endothelial Proliferation in the Spinal Cord, but Vascular Maturation Is Delayed

**Figure 2:**
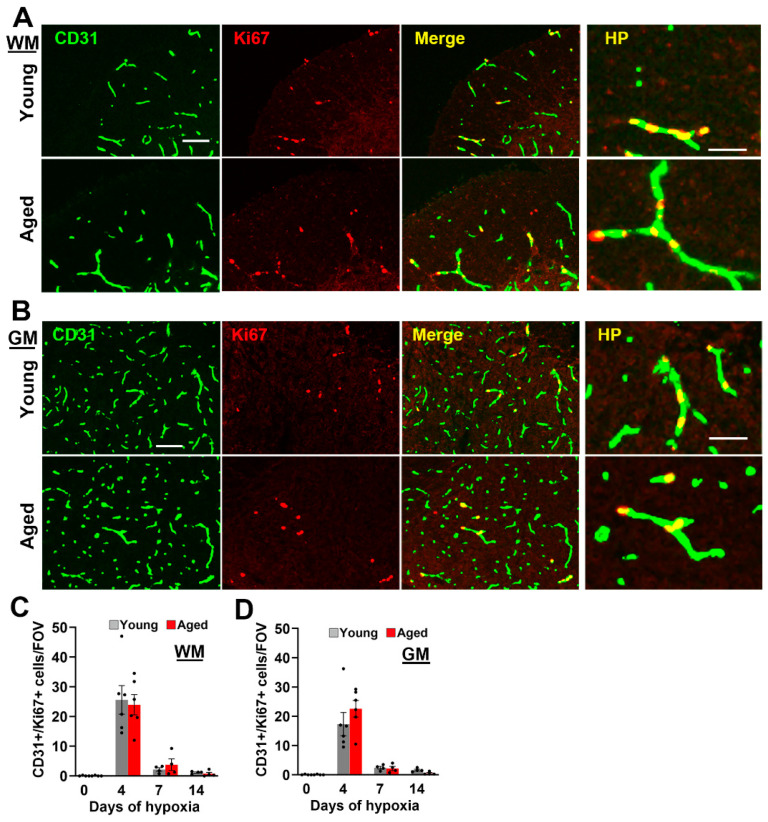
Evaluation of hypoxia-induced vascular remodeling in young and aged spinal cord WM (**A**) and GM (**B**). Frozen spinal cord sections taken from young (8–10 weeks) or aged (20 months) mice exposed to hypoxia (8% O_2_) for 4 days were stained for CD31 (AlexaFluor-488) and the proliferation marker Ki67 (Cy-3). Scale bar = 100 μm. High power (HP) images on the right highlight CD31/Ki67 co-localization. Scale bar = 50 μm. (**C**,**D**) Quantification of the number of proliferating endothelial cells (CD31+/Ki67+ cells)/FOV in WM (**C**) or GM (**D**) after 0, 4, 7, and 14 days hypoxia. Results are expressed as the mean ± SEM (*n* = 4–6 mice/group). Note that rates of endothelial proliferation in young and aged mice in both WM and GM were comparable.

**Figure 3:**
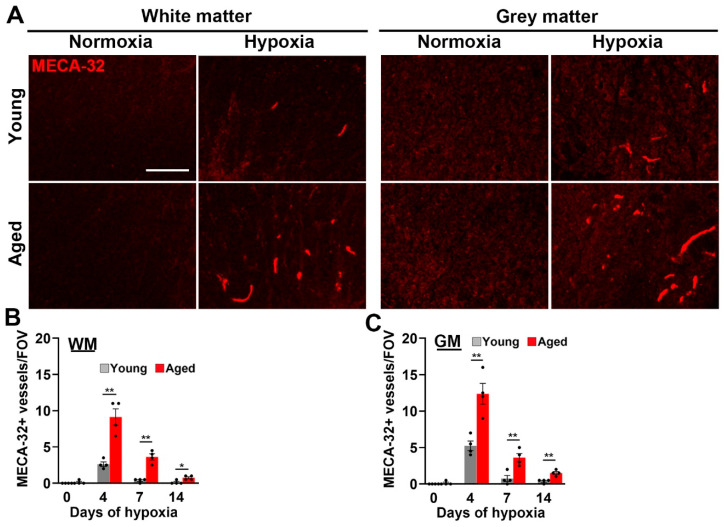
Evaluation of vessel maturation in young and aged spinal cord. (**A**) Frozen spinal cord sections taken from young (8–10 weeks) or aged (20 months) mice exposed to normoxia or hypoxia (8% O_2_) for 4 days were stained for the marker of immature brain endothelium MECA-32 (Cy-3). Scale bar = 100 μm. (**B**,**C**) Quantification of the number of MECA-32+ vessels/FOV in WM (**B**) or GM (**C**) after 0, 4, 7, and 14 days hypoxia. Results are expressed as the mean ± SEM (*n* = 4 mice/group). * *p* < 0.05, ** *p* < 0.01. Note that the number of MECA-32+ vessels peaked after 4 days and that the aged spinal cord showed a greater number of MECA-32+ vessels at all time-points, both in WM and GM.

As we previously showed that CMH promotes a strong vascular remodeling response in the spinal cord [7], we asked whether this is attenuated in aged mice by performing CD31/Ki67 dual-IF to quantify endothelial proliferation, a key early step in the process of angiogenesis. This revealed that, in both young and aged mice exposed to CMH, proliferating endothelial cells could be seen in spinal cord WM and GM (Figure 2A,B), and quantification showed that the rates of endothelial proliferation were largely equivalent in young and aged mice (Figure 2C,D). At both ages, endothelial proliferation in spinal cord blood vessels peaked after 4 days CMH, and then strongly declined at the later timepoints of 7 and 14 days CMH (Figure 2C,D). Interestingly, while rates of endothelial proliferation were similar in young and aged hypoxic spinal cord, the expression of the marker MECA-32, a marker of immature/remodelling cerebral blood vessels [21,22], was significantly enhanced in aged animals (*p* < 0.01 after 4 or 7 days CMH, both in WM and GM; Figure 3). These data are consistent with our recent findings in the brain [23] and suggest that in the spinal cord of aged mice, the maturation of remodeling blood vessels is significantly delayed (quantified in Figure 3B,C).

### 2.3. Microglial Activation in the Spinal Cord Is Greatly Increased by Age and CMH but, Paradoxically, the Microglial Vasculo-Protective Response Diminishes with Age

**Figure 4:**
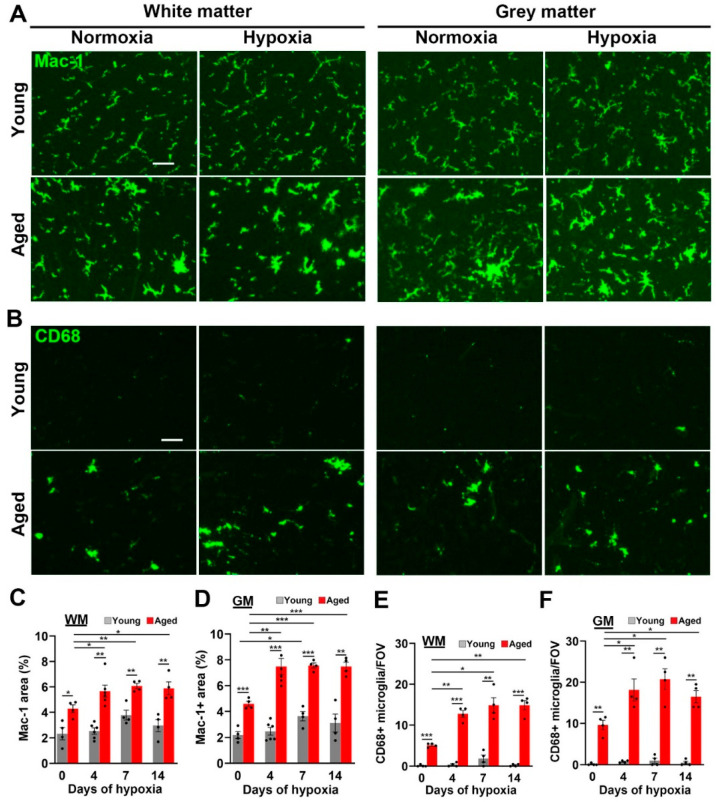
Microglia in the aged spinal cord show greatly enhanced levels of activation. (**A**,**B**) Frozen spinal cord sections taken from young (8–10 weeks) or aged (20 months) mice exposed to normoxia or hypoxia (8% O_2_) for 4 days were stained for Mac-1 (AlexaFluor-488) (**A**) or CD68 (AlexaFluor-488) (**B**). Scale bars = 50 μm. Quantification of the total Mac-1 area/FOV in WM (**C**) or GM (**D**), number of CD68+ cells/FOV in WM (**E**) or GM (**F**), after 0, 4, 7, and 14 days hypoxia. Results are expressed as the mean ± SEM (*n* = 4–6 mice/group). * *p* < 0.05, ** *p* < 0.01, *** *p* < 0.001. Note that microglia in the aged spinal cord are morphologically more activated and express higher levels of Mac-1 and CD68 at all time-points.

Next, we examined microglial activation responses in the spinal cords of young and aged mice. This is important for several reasons. Firstly, our prior work showed that microglial depletion led to much greater levels of hypoxia-induced vascular disruption both in the brain and spinal cord, demonstrating that microglia play an important vasculo-protective role during hypoxic exposure [7,24]. Secondly, in our recent study we observed that microglia in the aged brain are much more activated than in young mice under normoxic (control) conditions and, further, that they show a stronger activation response to CMH compared with young microglia [23]. However, paradoxically, despite this increased level of activation, microglia in aged brains were markedly deficient in their ability to aggregate around disrupted blood vessels [23]. To evaluate the impact of CMH and age on microglial activation in the spinal cord, we first performed Mac-1 IF. As shown in Figure 4A, most microglia in normoxic spinal cord WM and GM of young mice displayed the ramified morphology (small cell body with long processes) typical of resting microglia, and CMH had no obvious impact on microglial morphology. By contrast, even under normoxic conditions, microglia in aged spinal cord WM and GM displayed a much more activated morphology (large cell body and short thicker process extensions), and CMH further stimulated this level of activation. The quantification of the Mac-1 signal per field of view (FOV) confirmed that microglia in spinal cord WM and GM in aged mice were much more activated than in young mice, both under normoxic conditions (*p* < 0.05 and *p* < 0.001 in WM and GM respectively) and during CMH (*p* < 0.01 and *p* < 0.001 in WM and GM respectively, Figure 4C,D).

**Figure 5:**
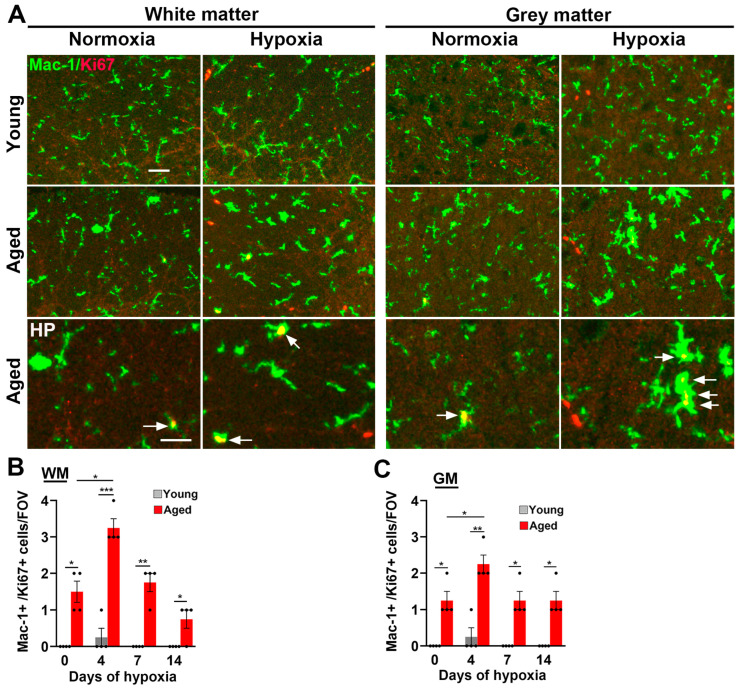
The microglial proliferation response to CMH is much greater in the aged spinal cord. (**A**) Frozen spinal cord sections taken from young (8–10 weeks) or aged (20 months) mice exposed to normoxia or hypoxia (8% O_2_) for 4 days were stained for Mac-1 (AlexaFluor-488) and the proliferation marker Ki67 (Cy-3). Scale bar = 50 μm. (**B**,**C**) Quantification of the number of Ki67+/Mac-1+ cells/FOV in the WM (**B**) and GM (**C**) after 0, 4, 7, and 14 days hypoxia. Results are expressed as the mean ± SEM (*n* = 4 mice/group). * *p* < 0.05, ** *p* < 0.01, *** *p* < 0.001. Note that microglia in the young spinal cord rarely proliferate, even in response to hypoxia, while those in the aged spinal cord show higher rates of proliferation at all time-points including normoxia and are further increased under hypoxic conditions (see arrows).

Analysis of another indicator of microglial activation, CD68 expression, which is a lysosomal marker of microglial priming [25], revealed a similar pattern. Specifically, while the number of CD68+ cells was extremely low in the normoxic spinal cords of young mice, and increased only marginally by CMH, spinal cords of aged mice (both WM and GM) contained a much higher number of CD68+ cells than young mice, both under normoxic conditions (*p* < 0.001 and *p* < 0.01 in WM and GM, respectively), and following exposure to CMH (*p* < 0.01; see Figure 4B and quantified in Figure 4E,F). Notably, while CMH had no obvious impact on CD68+ cell density in young mice, it significantly increased CD68+ cell density in aged mice (*p* < 0.01 and *p* < 0.05 in WM and GM, respectively). As microglial activation is associated with increased cell proliferation, we next examined microglial proliferation using Mac-1/Ki67 dual-IF. This revealed that, while very few proliferating microglia were present in young spinal cord WM or GM in normoxic or hypoxic conditions, microglial proliferation (denoted by arrows in Figure 5A) was evident in aged spinal cord even under normoxic conditions, and this number was further elevated by CMH, both in WM and GM (*p* < 0.05; Figure 5A–C).

**Figure 6:**
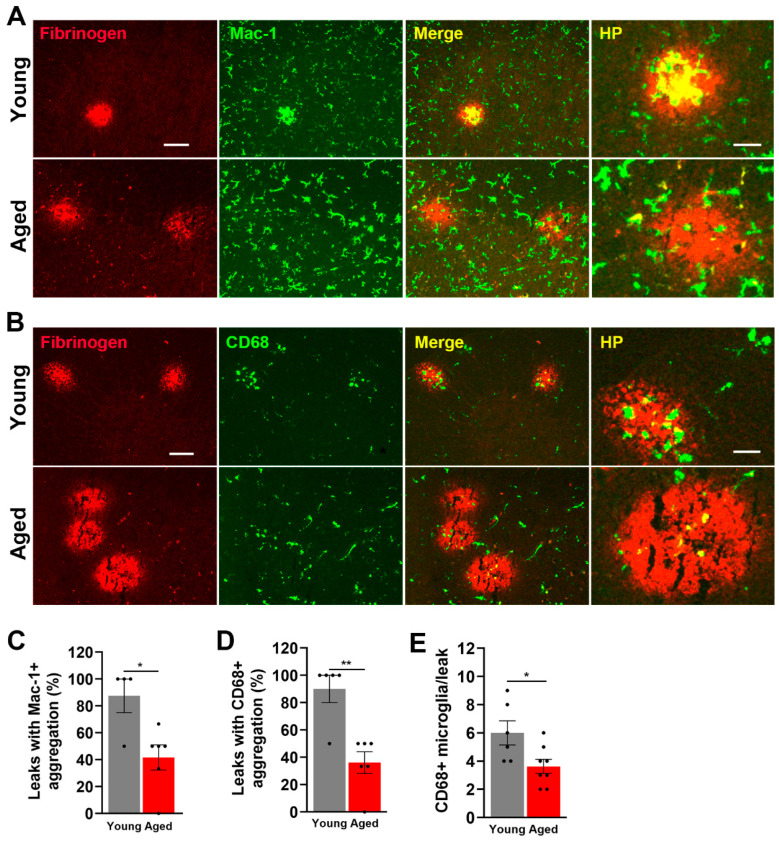
Microglia in the aged spinal cord show reduced aggregation around leaky blood vessels. Frozen spinal cord sections taken from young and aged mice exposed to hypoxia (8% O_2_) for 4 days were dual-stained for fibrinogen (Cy-3) and Mac-1 (AlexaFluor-488) (**A**) or fibrinogen (Cy-3) and CD68 (AlexaFluor-488) (**B**). Scale bars = 100 μm. Scale bar for high power (HP) images on the right = 50 μm. Quantification of the % of vascular leaks that are associated with Mac-1+ cellular aggregates (**C**) CD68+ cellular aggregates (**D**), or the number of CD68+ microglial cells per fibrinogen+ area (**E**) in young and aged spinal cord after 4 days hypoxia. Results are expressed as the mean ± SEM (*n* = 4–6 mice/group). * *p* < 0.05, ** *p* < 0.01. Note that microglial aggregation around fibrinogen+ vascular leaks in the aged spinal cord is markedly reduced.

As our prior analysis of hypoxic spinal cord in young mice demonstrated that fibrinogen-positive extravascular leaks are strongly associated with aggregation of activated microglia, and that these microglia play an important vasculo-protective response [24], we next evaluated the influence of age on this microglial response by performing fibrinogen/Mac-1 (Figure 6A) and fibrinogen/CD68 (Figure 6B) dual-IF on young and aged spinal cords. This revealed a marked deficit in the ability of aged microglia to accumulate around leaky spinal cord blood vessels. Specifically, while in young mice, most vascular disruptions were associated with cellular aggregates of Mac-1+ or CD68+ activated microglia, in the aged spinal cord, many vascular leaks were not associated with activated microglia, as shown by significant reduction in the number of vascular leaks associated with Mac-1+ (*p* < 0.05) or CD68+ (*p* < 0.01) cellular aggregates (shown in Figure 6A,B and quantified in Figure 6C,D). Furthermore, the number of CD68+ microglial cells per fibrinogen+ area was also reduced in aged spinal cord (6.00 ± 0.78 cells in young vs. 3.62 ± 0.47 cells in aged brain, *p* < 0.05, Figure 6E). Thus, although the aged spinal cord contains a much higher density of CD68+ activated microglia, paradoxically, the ability of microglia to migrate to and aggregate around leaky bloody vessels is severely compromised in the aged spinal cord.

### 2.4. Hypoxia-Induced Vascular Disruption in the Spinal Cord Is Associated with Loss of White Matter Myelin and Oligodendrocytes

**Figure 7:**
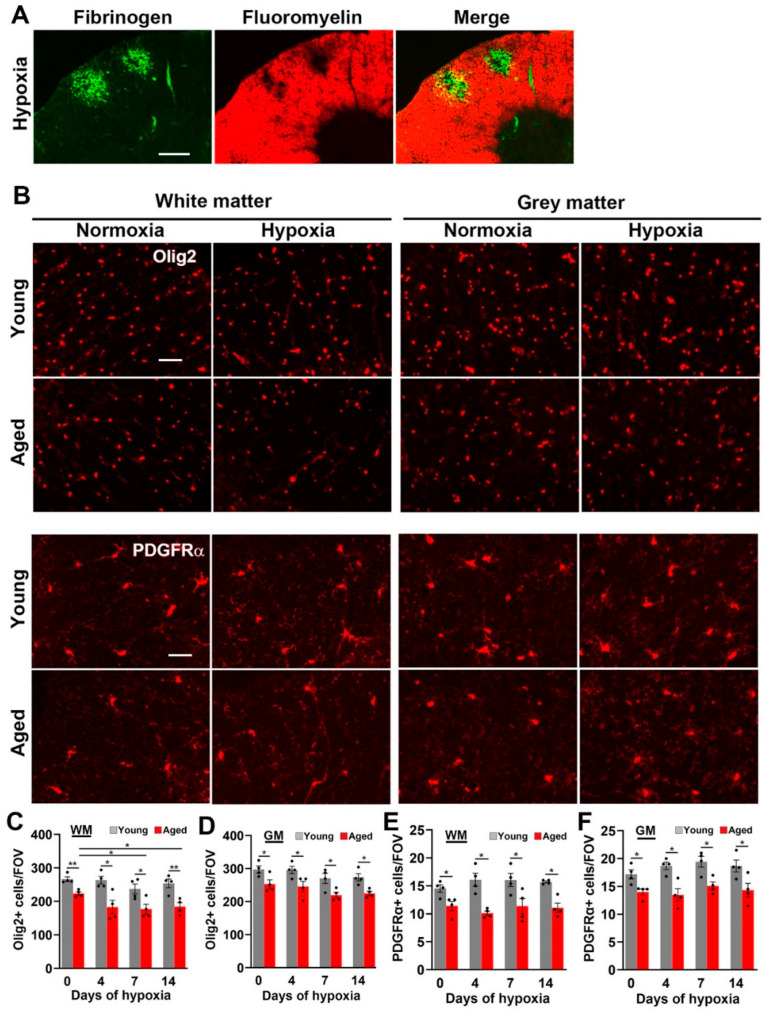
The impact of hypoxia and age on myelination in the spinal cord. (**A**) Frozen spinal cord sections taken from young mice exposed to hypoxia (8% O_2_) for 4 days were dual-stained for fibrinogen (AlexaFluor-488) and fluoromyelin (Cy-3). Scale bar = 100 μm. (**B**) Frozen spinal cord sections taken from young and aged mice exposed to normoxia or hypoxia (8% O_2_) for 4 days were stained for Olig2 (**top panel**) or PDGFRα (**lower panel**). Scale bars = 50 μm. Quantification of the cell density of Olig2+ cells in the WM (**C**) or GM (**D**) or the cell density of PDGFRα+ cells in the WM (**E**) or GM (**F**) during normoxic or hypoxic conditions. Results are expressed as the mean ± SEM (*n* = 4 mice/group). * *p* < 0.05, ** *p* < 0.01. Note that vascular leak results in marked loss of WM myelin and that the aged spinal cord contains significantly less Olig2+ oligodendroglial cells and PDGFRα+ oligodendrocyte precursor cells (OPCs).

As CMH induces a significant amount of vascular breakdown in white matter, we next examined whether this has any destructive effect on white matter myelin. As shown in Figure 7A, dual-IF with fibrinogen and fluoromyelin showed that vascular disruption was strongly associated with loss of myelin. To examine the impact of CMH on myelination at the cellular level, we next quantified the cell density of oligodendroglial lineage cells using the Olig2 marker (Figure 7B–D). This revealed several important points. Firstly, in keeping with previous reports, the overall density of Olig2+ cells in spinal cord WM and GM were very similar [26]. Secondly, the density of Olig2+ cells in the normoxic spinal cord was significantly reduced in aged mice both in WM (*p* < 0.01) and GM (*p* < 0.01), and this difference was maintained at all timepoints of CMH (*p* < 0.05). Thirdly, CMH exposure significantly reduced the density of Olig2+ cells in spinal cord WM in aged mice (*p* < 0.05), but not in young mice. We next examined how CMH influences the cell density of oligodendrocyte precursor cells (OPCs) by staining for platelet derived growth factor receptor alpha (PDGFRα). In a similar manner to Olig2 IF, this revealed that, first, the cell density of OPCs is very similar in WM and GM, and second, compared to the young, the aged spinal cord contains a reduced cell density of OPCs under normoxic conditions (*p* < 0.05 in WM and GM) and this effect was maintained at all CMH timepoints (*p* < 0.05). Interestingly, while CMH induced a slight non-significant upwards trend in OPC density in both WM and GM spinal cord in young mice, this response was absent in aged mice (Figure 7B,E,F), suggesting that OPCs in the spinal cords of young mice attempt to mount a regeneration response to replace lost myelin, but this ability is lost in aged mice.

## 3. Discussion

These studies demonstrate that hypoxia triggers greater pathology in the aged spinal cord compared to the young, as determined by the following readouts: (i) 3–4-fold greater vascular disruption defined by extravascular fibrinogen leak, (ii) delayed maturation of newly formed blood vessels, as indicated by prolonged MECA-32 expression, (iii) markedly increased microglial activation, which paradoxically correlated with reduced vasculo-protective function, and (iv) significantly reduced density of WM oligodendroglial cells. Importantly, while hypoxic-induced vascular disruption was closely associated with loss of spinal cord WM myelin in both young and aged mice, the density of oligodendroglia and their precursors throughout the aged spinal cord was significantly lower than the young, both under baseline normoxic conditions and after CMH. Taken together, these results demonstrate that spinal cord blood vessels in aged mice are far more susceptible to the destructive effects of hypoxia than young mice, which may be explained in part by the reduced vasculo-protective activity of chronically activated microglial cells. Our findings provide insight into the potential role that hypoxia may play in increasing vulnerability to age-related spinal cord diseases such as ALS and raise the possibility that the optimization of microglial vasculo-protective function may offer clinical benefit in preventing or treating age-related spinal cord conditions.

### 3.1. The Impact of Aging on Spinal Cord Blood Vessels

Our finding of increased hypoxia-induced vascular disruption in the aged spinal cord is consistent with recent observations in the brain [23]. Several studies have highlighted age-related decreased vascular integrity in the brain [27,28], and our results suggest that vascular integrity of the spinal cord shows similar deterioration. Of note, while the aged spinal cord was more susceptible to hypoxia-induced vascular leak, the endothelial proliferation response to hypoxia was no different between young and aged spinal cord, though reminiscent of the brain, maturation of newly formed blood vessels was slowed considerably, as shown by prolonged expression of MECA-32, a marker of immature CNS endothelium [23]. BSCB disruption has been reported in several disease conditions of the spinal cord, including ALS, MS, and spinal cord injury [9,10,11,12], suggesting that reduction in vascular integrity may be an important factor influencing disease pathogenesis. Interestingly, previous studies have suggested that BSCB integrity is generally less than BBB integrity, and that this might be a result of reduced pericyte coverage concordant with the attenuated expression of endothelial tight junction proteins in spinal cord vessels [29]. In support of this idea, genetically modified mice with reduced pericyte coverage of spinal cord blood vessels display enhanced vascular permeability, correlating with the destruction of motor neurons. Based on these findings, it was suggested that a reduction in spinal cord pericytes may predispose to heightened vascular permeability and predispose to the development of ALS [29]. However, contrary to this, a recent study of post-mortem spinal cords from ALS patients showed that, while vascular leak and neuronal pathology, as measured by TAR DNA-binding protein 43 (TDP-43) pathology, were both present in spinal cord tissue, these events were not spatially correlated [12]. Based on these observations, the authors concluded that BSCB disruption and neuronal pathology are independent pathologies in ALS. However, an alternative possibility is that BSCB disruption leads to the destruction of motor neurons in the vicinity of vascular leak, but that, over time, the vascular leaks are repaired while the neuronal damage is irreversible. This sequence of events would lead to a disconnect of BSCB disruption and neuronal pathology at later timepoints, resulting in exactly the type of pattern described in the post-mortem study.

### 3.2. Comparison of Vascular Responses in White Matter and Grey Matter

One of the goals of this project was to determine if blood vessels in spinal cord WM and GM show any obvious differences in their response to hypoxic insult. In fact, both the density of vascular leaks and rate of endothelial proliferation were remarkably similar in WM and GM, within the young and aged spinal cord. While, at first glance, this may appear that WM and GM have similar vascular responses to hypoxia, it is important to consider that because GM vessel density is roughly four times greater than WM [13,19,20], it points to the much greater propensity of WM blood vessels to launch a vascular remodeling response (which includes BSCB disruption) to hypoxia. What accounts for this heightened responsiveness of WM blood vessels is currently unknown, though we propose that because WM vessel density is so much less than GM, taken with the high metabolic rate of WM oligodendrocytes, the hypoxic drive is much higher in this region, leading to an enhanced vascular remodeling response.

### 3.3. The Impact of CMH on Myelin and OLG in Young and Aged Spinal Cord

Previous studies have shown that the myelin-forming cells of the CNS, oligodendrocytes, are acutely sensitive to the destructive effects of hypoxia, both in the young and the aged [30,31,32,33]. Our findings add to this body of data by showing that, not only is the density of oligodendroglial cells and their precursors lower in the spinal cord of aged mice under normoxic conditions but, superimposed on this, CMH reduced the density of WM oligodendroglial cells in aged brains but not in young brains. This outcome could be the result of an increased sensitivity of aged oligodendrocytes to hypoxia but may also be partly explained by a more active regeneration response in the young, as seen by the trend towards enhanced density of OPCs in young brain, but not in aged. In addition to the widespread effects of hypoxia and age on myelin and oligodendrocytes throughout the whole spinal cord, we also demonstrated acute myelin loss in the close vicinity of BSCB breakdown and fibrinogen deposition, in keeping with recent evidence linking fibrinogen leak directly to oligodendrocyte cell death [34].

### 3.4. Microglia in Aged Spinal Cord Show Greatly Enhanced Activation but Reduced Vasculo-Protection

It comes as no surprise that the impact of age and hypoxia on microglial behavior in the spinal cord shows a lot of similarity to microglia in the brain. Specifically, under normoxic conditions, microglia in the aged spinal cord were found to be much more activated than the young under non-challenged normoxic conditions, and while CMH has little impact on microglial activation state in the young spinal cord, in aged spinal cord, CMH strongly enhances activation state. Most importantly, from a functional viewpoint, just as in aged brain, the heightened activation state of microglia in the aged spinal cord correlates closely with a greatly reduced ability to aggregate round and repair disrupted blood vessels [23]. These findings highlight hypoxia as a potential contributor in the pathogenesis of age-related spinal cord diseases such as ALS. Perhaps more importantly, they suggest that a greater mechanistic understanding of why aged microglia lose their vasculo-protective function could open the door to novel therapeutic strategies aimed at tightening BSCB integrity in the aged as a means of preventing or treating age-related spinal cord disease.

## 4. Materials and Methods

### 4.1. Animals

The studies described were reviewed and approved by the Institutional Animal Care and Use Committee at San Diego Biomedical Research Institute (SDBRI). Wild-type female C57BL6/J mice, obtained from Jackson Laboratories and the NIH National Institute on Aging (NIA) Aged Rodent Colony, were maintained under pathogen-free conditions in the closed breeding colony of SDBRI.

### 4.2. Chronic Hypoxia Model

Female C57BL6/J mice, 8–10 weeks (young) and 20 months (aged), were housed 4 to a cage, and placed into a hypoxic chamber (Biospherix, Redfield, NY, USA) maintained at 8% O_2_ for periods up to 14 days. Littermate control mice were kept in the same room under similar conditions except that they were kept at ambient sea-level oxygen levels (normoxia, approximately 21% O_2_ at sea-level) for the duration of the experiment. Every few days, the chamber was briefly opened for cage cleaning and food and water replacement as needed.

### 4.3. Immunohistochemistry and Antibodies

Immunohistochemistry was performed on 10 µm frozen sections of cold phosphate buffer saline (PBS) perfused spinal cord tissues as described previously [35]. Rat monoclonal antibodies from BD Pharmingen (La Jolla, CA, USA) reactive for the following antigens were used in this study: CD31 (clone MEC13.3; 1:300), MECA-32 (1:100), Mac-1 (clone M1/70; 1:50), and CD68 (clone FA-11; 1:2000). The hamster anti-CD31 (clone 2H8; 1:500) was obtained from Abcam (Cambridge, MA, USA) and the rat anti-TER-119 (1:2000) and goat anti-PDGFRα (1:1000) were obtained from R&D systems. Rabbit antibodies reactive for the following proteins were also used: Ki67 (1:4000 from Novus Biologicals, Centennial, CO, USA), Olig2 (1:1000 from Novus Biologicals), and fibrinogen (1:1500 from Millipore, Temecula, CA, USA). Fluoromyelin red (1:50) was obtained from Invitrogen (Carlsbad, CA, USA). Secondary antibodies used (all at 1:500) included Cy3-conjugated anti-rabbit, anti-rat, and anti-goat and Cy5-conjugated anti-rabbit from Jackson Immunoresearch, (West Grove, PA, USA) and Alexa Fluor 488-conjugated anti-rat, anti-hamster, and anti-rabbit from Invitrogen.

### 4.4. Image Analysis

Images were taken using a 5x 10x or 20x objective on a Zeiss Imager M1.m fluorescent microscope (Thornwood, NY, USA). For each antigen in all analyses, images of at least three randomly selected areas were taken at 5x, 10x or 20x magnification per tissue section and three sections per brain analyzed to calculate the mean for each animal (*n* = 4–6 mice per group). For each antigen in each experiment, exposure time was set to convey the maximum amount of information without saturating the image and was maintained constant for each antigen across the different experimental groups. The number of vascular leaks or MECA-32+ vessels per field of view (FOV) was quantified by capturing images and performing manual counts of the number of vessels showing extravascular leaked fibrinogen or MECA-32 respectively. The number of activated microglia or cell density of Olig2+ oligodendroglial cells or PDGFRα+ oligodendrocyte precursor cells (OPCs) were quantified by performing manual counts of the number of CD68+, Olig2+ or PDGFRα+ cells per FOV. The total Mac-1+ area fluorescent signal per FOV was measured and analyzed using NIH Image J software, version 1.53t. Endothelial and microglial proliferation were quantified by counting the number of CD31/Ki67 or Mac-1/Ki67 dual-positive cells per FOV, respectively. The number of fibrinogen+ leaky vessels showing Mac-1+ or CD68+ microglial accumulation was quantified by capturing images and performing manual counts. Each experiment was performed with 4–6 different animals per condition, and the results expressed as the mean ± SEM. All data displayed normal distribution and statistical significance was assessed using one-way analysis of variance (ANOVA) followed by Tukey’s multiple comparison post hoc test or Student’s *t* test in which *p* < 0.05 was defined as statistically significant.

## Data Availability

The datasets used and/or analyzed during the current study are available from the corresponding author upon reasonable request.
